# Effect of metformin use on the risk and prognosis of endometrial cancer: a systematic review and meta-analysis

**DOI:** 10.1186/s12885-018-4334-5

**Published:** 2018-04-18

**Authors:** Danxia Chu, Jie Wu, Kaili Wang, Mengling Zhao, Chunfang Wang, Liuxia Li, Ruixia Guo

**Affiliations:** Department of Gynecology, The First Affiliated Hospital of Zhengzhou University, NO.1, JianShe East Road, Zhengzhou, 450052 Henan China

**Keywords:** Endometrial cancer, Metformin, Risk, Prognosis, Meta-analysis

## Abstract

**Background:**

Previous studies have suggested that metformin may be useful for preventing and treating endometrial cancer (EC), while the results have been inconsistent. This systematic review and meta-analysis aimed to investigate the association between metformin use and risk and prognosis of patients with EC.

**Methods:**

PubMed, Embase, and the Cochrane Library databases were searched for observational studies evaluating the effect of metformin on EC prevention or treatment. The odds ratio (OR) was used for analyzing risks, and the hazard ratio (HR) was used for analyzing survival outcomes. A random-effects model was used for data analysis.

**Results:**

Seven studies reported data on EC risk. The pooled results suggested that metformin was not significantly associated with a lower risk of EC [OR = 1.05, 95% confidence interval (CI) 0.82–1.35, P = 0.70]. For patients with diabetes, metformin showed no advantage in reducing the EC risk compared with other interventions (OR = 0.99, 95% CI 0.78–1.26, P = 0.95). Further, seven studies were included for survival analysis. The pooled data showed that metformin could significantly improve the overall survival of patients with EC (HR = 0.61, 95% CI 0.48–0.77, *P* < 0.05) and reduce the risk of EC recurrence (OR = 0.50, 95% CI 0.28–0.92, *P* < 0.05) Finally, we noted metformin was associated with significantly improving the overall survival of EC patients among diabetes (HR = 0.47; 95%CI 0.33–0.67, *P* < 0.05).

**Conclusions:**

This meta-analysis did not prove that metformin was beneficial for preventing EC. However, metformin could prolong the overall survival of patients with EC and reduce their risk of cancer relapse.

**Electronic supplementary material:**

The online version of this article (10.1186/s12885-018-4334-5) contains supplementary material, which is available to authorized users.

## Background

Endometrial cancer (EC), a tumor originating from the endometrium, is a major cause of morbidity and mortality in women. Hyperplastic endometrium may be a result of exposure to unopposed estrogen, leading to the progression of cancer. It is the most common malignancy of the female genital tract in the United States, with approximately 54,870 new cases and 10,170 related deaths in 2015 [1]. The incidence of EC is lower in developing countries compared with developed countries. However, EC was associated with higher cancer mortality and poor prognosis in developing countries [1–3]. Further, despite advances in the treatment of EC, the prognosis for stages III–IV EC remains poor [4].

Various adjuvant medications have been suggested for preventing and treating EC, including aromatase inhibitors [5], aspirin [6], statins [7], hormone therapy [8], and metformin [9, 10]. Metformin has several advantages in addition to its anticancer activity. First, it is a first-line pharmacologic treatment for patients with type 2 diabetes mellitus [11]. Second, in addition to metformin use for diabetes, it is also safely prescribed for various nondiabetic conditions, including polycystic ovarian syndrome [12], primary prevention of type 2 diabetes mellitus and cardiovascular diseases [13, 14], and obesity control [15]. Finally, metformin is readily available worldwide at low cost.

Type 2 diabetes mellitus is a well-established risk factor for EC [16, 17]. Insulin resistance has been suggested to be one of the critical biological processes that contribute to EC [18, 19]. Approximately 30% of patients with EC have type 2 diabetes mellitus, and up to 36% have undiagnosed insulin resistance [20]. Metformin use could reduce the risk of type 2 diabetes mellitus and delay its progression. It reduces insulin resistance by increasing insulin receptor tyrosine kinase activity, enhancing glycogen synthesis, and promoting the recruitment and increasing the activity of glucose transporter type 4 [21]. Moreover, it affects endometrial maturation, proliferation, and implantation process [22–24]. Finally, the risk of EC is increased in women who have higher endogenous estrogen levels [25], and metformin has been reported to hinder estrogen-mediated endometrial proliferation [26].

Several studies have reported that metformin is a promising intervention for preventing and treating EC. However, these studies had conflicting results, and no relevant meta-analyses have been conducted. Therefore, this systematic review and meta-analysis was performed to evaluate the effectiveness of metformin for the risk and survival outcomes in patients with EC.

## Methods

### Search strategy

This meta-analysis was performed according to the Preferred Reporting Items for Systematic Reviews and Meta-Analysis guidelines (Additional file 1) [27]. PubMed, Embase, and the Cochrane Library databases were searched for eligible studies between 1980 and July 2016. The following key words and medical terms were used for the literature search: (metformin OR glucophage OR dimethylbiguanide OR dimethylguanylguanidine) AND (endometrial cancer OR endometrial carcinoma OR endometrial hyperplasia OR endometrial proliferation OR endometrial thickness). The language was limited to English. Manual searching was also conducted on the reference lists of included studies and reviews for potentially relevant studies.

### Inclusion criteria

Articles were included in the study if they met the following criteria: (1) used metformin for preventing or treating EC; (2) evaluated the incidence of EC or survival outcomes; the survival endpoints were overall survival (OS), recurrence-free survival (RFS)/disease-free survival, or the recurrence rate; (3) directly reported the effect estimates of odds ratio (OR), hazard ratio (HR), or relative risk (RR); (4) indirectly reported data allowing for the calculation of these effect estimates; and (5) the study with observational design. Abstracts, unpublished data, and studies not published in English were excluded.

### Data collection and quality assessment

Two reviewers independently examined the included studies for eligibility and extracted the data. Any disagreement was resolved by consensus. The following information from each included study was extracted into standardized tables: author, publication year, region, study design, sample size, age, patient characteristics, EC incidence, percentage of metformin use in DM patients, proportion of patients with diabetes, reported effect estimates, degree of adjustment, and study period. When multiple studies for the same cohort were found, the most comprehensive or most recent data were used. The same reviewers independently evaluated the risk of bias using the Newcastle–Ottawa Scale [28]. This scale assigned 0–9 points based on three items: selection, comparability, and outcome assessment. Studies with 0–3, 4–6, and 7–9 points were classified as low-, medium-, and high-quality studies, respectively.

### Statistical analysis

ORs and their associated 95% confidence intervals (CIs) were used as the effect measures for the outcome of EC risk. HRs and associated 95% CIs were used as the effect measures for the survival outcomes. Adjusted ORs or HRs were preferred for the analyses. However, if the adjusted effect estimates were not directly presented, they were calculated from the crude data available. HR was considered to be equivalent to RR in cohort studies. Given the low incidence of EC, HRs could be assumed to be accurate estimates of ORs. When a study presented only Kaplan–Meier curves, HRs and 95% CIs were calculated based on published methods [29]. A fixed-effects model was used for the data combination of different subgroups in a single study. The result from a random-effects meta-analysis is more conservative than that from a fixed-effects model. Thus, the between-study results were pooled using the random-effects model proposed by DerSimonian and Laird [30]. The heterogeneity between studies was evaluated with Q and *I*^2^ statistics [31]. *I*^2^ values between 0% and 25% were designated as a low level, values more than 25% as a moderate level, and values more than 75% as a high level of heterogeneity. The heterogeneity was explored by subgroup, meta-regression, and sensitivity analyses. The publication bias was examined visually by the symmetry of funnel plots and statistically by Egger’s or Begg’s tests [32, 33]. All statistical analyses were performed using Stata software (version 12.0, Stata Corporation, TX, USA). All statistical tests were two sided. A *P* value less than 0.05 was considered statistically significant.

## Results

### Study selection

**Fig. 1:**
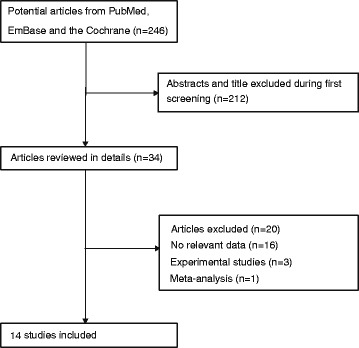
Flow diagram of the study selection process

The PRISMA flow diagram of the identification and selection of studies is shown in Fig. 1. From a total of 246 publications (141 from PubMed, 97 from Embase, and 8 from the Cochrane Library), duplicates were removed and irrelevant studies or those without sufficient data discarded. Finally, 13 studies were pooled in the meta-analysis, including 7 studies on the risk of EC [34–40] and 7 studies on the survival outcomes of EC [9, 10, 41–45].

### Study characteristics and quality evaluation

The characteristics of the seven studies on EC risk are shown in Table 1, including two case–control studies [34, 39], four retrospective studies [36–38, 40], and one prospective study [35]. These studies were all published between 2013 and 2017. Two studies were conducted in the United States [35–37], three in Europe [34, 39, 40], and one in China [38]. The sample size ranged from 7861 to 4,478,921. Six studies reported the adjusted ORs [34–36, 38–40], and one study reported only the crude data [37]. The incidence of EC ranged from 0.1% to 14.3% of included studies. The features of the studies on EC prognosis are shown in Table 2. All of the studies were retrospectively designed, including five studies in the United States [9, 10, 41, 43, 44] and two studies in Europe [42, 45]. The sample size ranged from 107 to 1303. Except for a study that included only cases of advanced EC [44], most of the studies primarily included patients with early-stage EC (70%–82%). The proportion of patients with diabetes ranged from 17% to 100%. Hall et al. only presented crude data on recurrence rates [10], but the other studies reported adjusted data. The quality appraisal of the included studies is summarized in Additional file 2. All studies were of high quality, with a score of 8–9.

**Table 1:** Characteristics of included studies on the risk of endometrial cancer

Author (year)	Region	Design	No. of patients	Mean/Median age (year)	EC incidence	Percentage of metformin use in DM patients (%)	Measurement of outcome	Adjusted variables	Subgroups	Study period
Becker et al. (2013) [34]	UK	Case–control	17,878	63	14.3%	51.5	Adjusted OR	BMI, smoking, DM	Metformin intensity, DM state	1995–2012
Luo et al. (2014) [35]	USA	Prospective	88,107	63	1.4%	12.5	Adjusted OR	Age, ethnicity, education, smoking, PA, alcohol, hormone use, parity, contraception, BMI	BMI	1993–1998
Ko et al. (2015) [36]	USA	Retrospective	541,128	52	0.1%	84.4	Adjusted HR	Age, Charlson index, fibroid, infertility, PCOS, DM, hypertension, endometrial hyperplasia, connective tissue disease, oral contraceptive, HRT, ultrasound	Age, DM state, PCOS, EH	2000–2011
Soffer et al. (2015) [37]	USA	Retrospective	66,778	55	0.5%	40.3	Unadjusted OR	None	None	1998–2004
Tseng et al. (2015) [38]	China	Retrospective	4,478,921	56	0.6%	40.3	Adjusted HR	Age, hypertension, COPD, stroke, heart disease, obesity, metabolic profiles, various drugs	Duration, dose	1998–2002
Franchi et al. (2016) [39]	Italy	Case–control	7861	> 40	4.8%	36.1	Adjusted OR	Charlson index, medical conditions, prescription of selected drugs, antidiabetic drugs	Duration	2002–2007
Arima et al. (2017) [40]	Finland	Retrospective	92,366	> 40	0.6%	63.8	Adjusted HR	Age, duration of DM and use at any time of other forms of medication.	None	1996–2011

*BMI* Body mass index, *COPD* chronic obstructive pulmonary disease, *DM* diabetes mellitus, *EC* endometrial cancer, *EH* endometrial hyperplasia, *HR* hazard ratio, *HRT* hormone replacement therapy, *OR* odds ratio, *PA* physical activity, *PCOS* polycystic ovary syndrome, *UK* United Kingdom, *USA* the United States of America

**Table 2:** Characteristics of included studies on the survival outcomes of endometrial cancer

Author (year)	Region	Design	Percentage of stages I–II, %	No. of patients	Age (year)	Diabetic patients, %	Adjusted variables	Outcomes	Subgroups	Follow-up duration
Ko et al. (2014) [41]	USA	Retrospective	82	363	63	100	Age, stage, grade, histology, adjuvant treatment	TTR, RFS, OS	None	2005–2010
Nevadunsky et al. (2014) [9]	USA	Retrospective	75	985	64	75	Age, clinical stage, grade, chemotherapy, radiation, hyperlipidemia	OS	Diabetes	1999–2009
Lemanska et al. (2015) [42]	Poland	Retrospective	75	107	64	64	Age, diabetes, hypertension, BMI, glucose level, grade, stage, hysterectomy, radiation, endometrial cancer type	OS	Histological type	2002–2010
Ezewuiro et al. (2016) [44]	USA	Retrospective	0	349	63	17	study site, BMI, race, age, stage	OS, recurrence	Diabetes	1992–2011
Hall et al. (2016) [10]	USA	Retrospective	79	351	58	18	None	Recurrence	None	2005–2011
Al Hilli et al. (2016) [43]	USA	Retrospective	81	1303	65	21	Age, BMI, smoking, ASA score, cardiopulmonary state, various tumor features, surgery, adjuvant therapy	OS, PFS	Histological type, diabetes	1999–2008
Seebacher et al. (2016) [45]	Austria	Retrospective	70	465	65	19	Age, tumor stage, grade, histological subtype	CSS, OS, RFS, recurrence	None	1995–2011

*ASA* American Society of Anesthesiologists, *BMI* body mass index, *CSS* cancer-specific survival, *EC* endometrial cancer, *OS* overall survival, *PFS* progression -free survival, *RFS* recurrence-free survival, *TTR* time to recurrence, *USA* the United States of America

### Metformin and the risk of EC

**Fig. 2:**
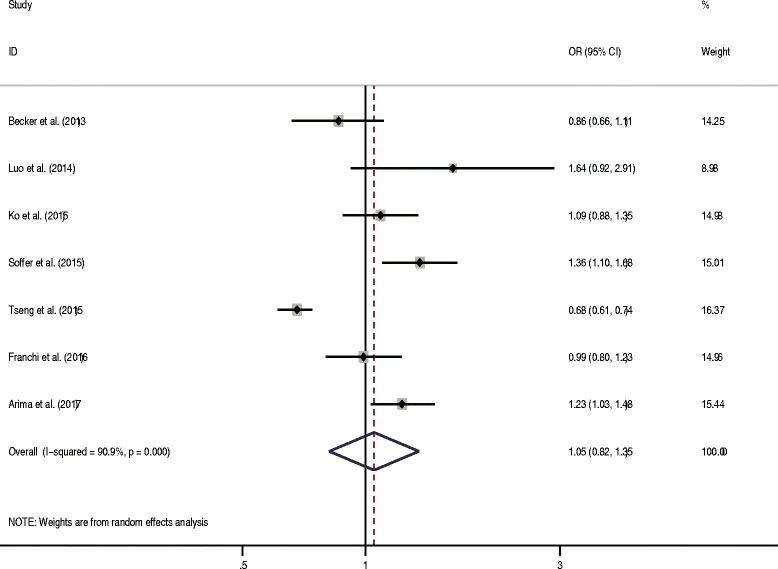
Forest plot of the association between metformin use and risk of endometrial cancer

**Fig. 3:**
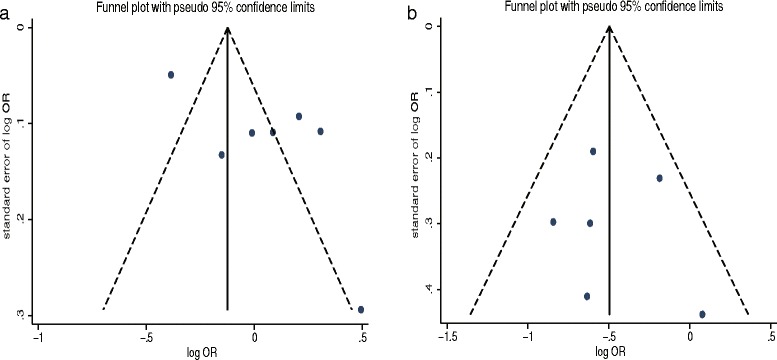
Funnel plot of the included studies: **a** Studies presenting the association between metformin use and risk of endometrial cancer (EC). **b** Studies presenting an adjusted hazard ratio for the association between metformin use and overall survival of EC

Seven studies were eligible for the meta-analysis. Becker et al. analyzed the risk based on different metformin prescriptions (1–24 and ≥ 25) [34]. The data were first pooled in a fixed-effects model. Six studies presented the adjusted ORs. The pooled data showed that metformin use was not associated with the risk of EC (OR = 1.05, 95% CI 0.82–1.35, *P* = 0.70) (Fig. 2). High heterogeneity was shown among the studies (*I*^2^ = 90.9%, *P* < 0.05). In the sensitivity analysis, the exclusion of any single study did not markedly alter the overall effect size, and in stratified analyses, the overall effect was not significantly changed for the subgroups by geographic region (USA, Europe, or Asia) and study design (case–control, prospective, or retrospective). However, no heterogeneity was detected in two European case–control studies (*I*^2^ = 0). In the meta-regression analysis, the sample size could not explain the source of heterogeneity (*P* > 0.05). The funnel plot appeared to be symmetrical (Fig. 3a). Notably, a significant publication bias was revealed by the Egger’s test (*P* < 0.05), but not by the Begg’s test (*P* = 1.00). The conclusions were not changed after adjustment for publication bias by using the trim and fill method [46].

**Fig. 4:**
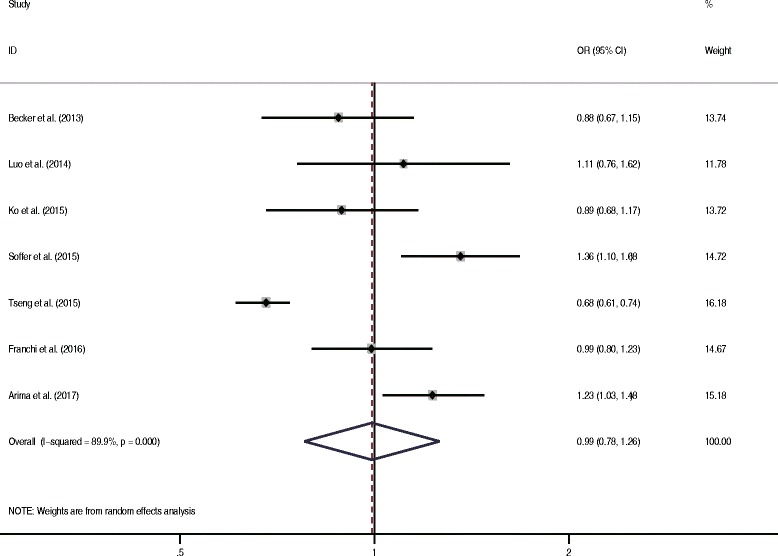
Forest plot of the association between metformin use and risk of endometrial cancer among patients with diabetes

The association between metformin and EC risk was further investigated in patients with diabetes. Franchi et al., Tseng et al. and Arima et al. included only patients with type 2 diabetes mellitus [37–40]. Three other studies reported data for subpopulations with type 2 diabetes mellitus [34–36]. The pooled data showed that patients with diabetes using metformin did not have a substantially lower risk of EC compared with those receiving other interventions (OR = 0.99, 95% CI 0.78–1.26, *P* = 0.95) (Fig. 4). However, high heterogeneity was detected (*I*^2^ = 89.9%, *P* < 0.05).

### Metformin and OS of EC

**Fig. 5:**
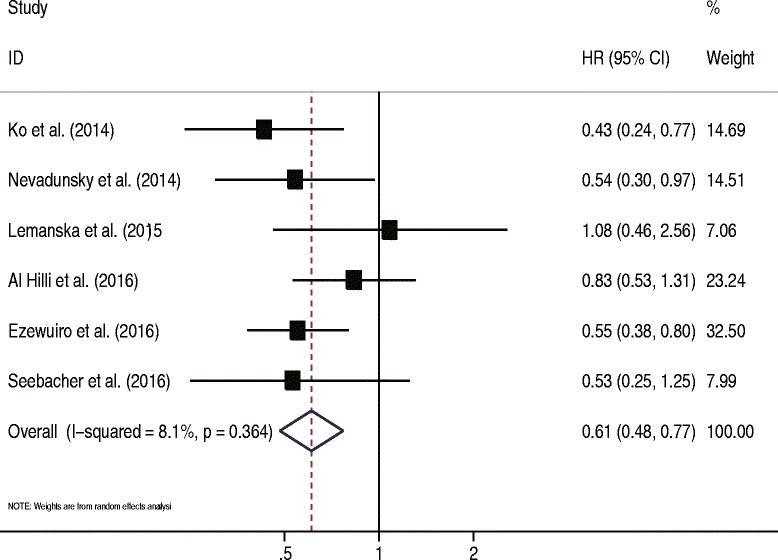
Forest plot of the association between metformin use and overall survival of endometrial cancer

Six retrospective studies were included in this analysis. Ezewuiro et al. and Al Hilli et al. separately reported data for patients with diabetes not using metformin and those without diabetes [43, 44]. The data from the subgroups within a single study were first pooled using a fixed-effects model. The pooled data showed that metformin use in patients with EC was significantly associated with longer OS compared with patients with EC not using metformin (HR = 0.61, 95% CI 0.48–0.77, *P* < 0.05) (Fig. 5). Low heterogeneity was identified, which was not significant (*I*^2^ = 8.1%, *P* = 0.36). No single study markedly changed the overall effect in the sensitivity analysis. In the subgroup analysis, the results were not significant for two European studies (HR = 0.74, 95% CI 0.37–1.49, *P* = 0.41), but were significant for the four studies conducted in the USA (HR = 0.59, 95% CI 0.45–0.76, *P* < 0.05). When stratified by the percentage of patients with type 2 diabetes mellitus (< 50% vs ≥50%), the overall effect had no substantial change. The meta-regression analysis revealed no significant role of sample size (*P* = 0.81), proportion of stages I–II patients (*P* = 0.88), or percentage of patients with type 2 diabetes mellitus (*P* = 0.84) to account for the heterogeneity. The funnel plot seemed to be symmetrical (Fig. 3b). No significant publication bias was shown by the Egger’s test (*P* = 0.78) or the Begg’s test (*P* = 0.45).

**Fig. 6:**
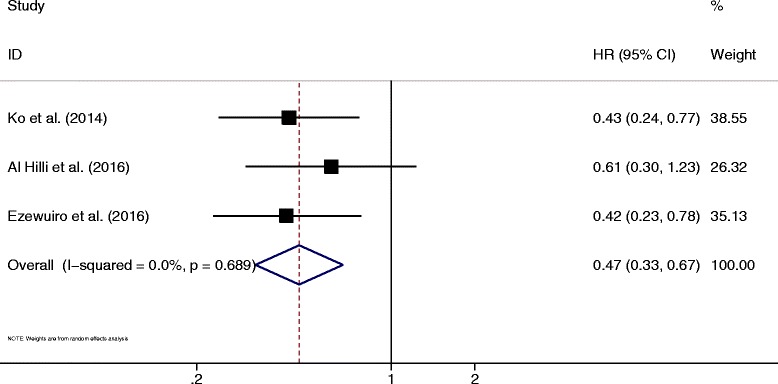
Forest plot of the association between metformin use and overall survival of endometrial cancer among patients with diabetes

The efficacy of metformin was further investigated among patients with EC having diabetes. Three studies reported relevant data. Metformin use was significantly associated with improved OS compared with other antidiabetic regimens (OR = 0.47, 95% CI 0.33–0.67, *P* < 0.05) (Fig. 6). No heterogeneity was detected (*I*^2^ = 0%, *P* = 0.69).

### Metformin and recurrence of EC

**Fig. 7:**
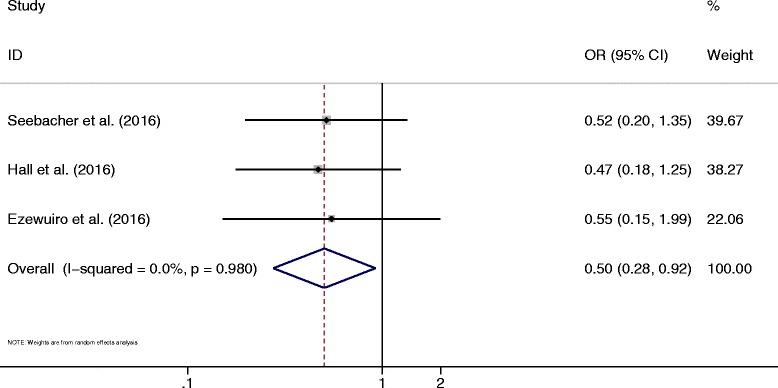
Forest plot of the association between metformin use and recurrence of endometrial cancer

Three studies reported data on the recurrence of EC [10, 44, 45]. The pooled results suggested that metformin use in patients with EC did not significantly reduce the risk of recurrence (OR = 0.50, 95% CI 0.28–0.92, *P* < 0.05) (Fig. 7). No heterogeneity was identified (*I*^2^ = 0%, *P* = 0.98).

## Discussion

This meta-analysis on the prevention of EC with metformin included 7 studies and a total of 5,293,039 participants. The pooled data suggested that the use of metformin could not substantially prevent the development of EC. When analyzing the subgroup of patients with diabetes, who were at a higher risk of EC, a significant protective effect of metformin against EC still could not be detected compared with patients with diabetes treated with other antidiabetic therapies. Differences in the duration of use and dose of metformin might have limited the statistical power of this study. The protective effects of metformin might be time and dose dependent [47]. However, most included studies failed to conduct dose-escalation analyses. Further, this meta-analysis also comprised 7 studies with a total of 3923 patients with EC who were treated with metformin. It was found that metformin could substantially improve the OS and reduce the risk of recurrence. The benefit for OS remained significant for the subgroup of patients with diabetes.

A previous meta-analysis based on 19 studies and illustrated effects of metformin on reversal of atypical endometrial hyperplasia, cellular proliferation biomarkers expression and overall survival. Further, they point out metformin could reverse atypical endometrial hyperplasia to normal endometrial histology, reduction of cell proliferation biomarkers, and improvement of OS. However, mostly investigated outcomes focused on precancerous indicators, while the risk of certain cancer was not evaluated [48]. The study conducted by Perez-Lopez et al. suggested metformin therapy was associated with a reduced risk of overall mortality in T2DM women with EC. Whereas this study focused on patients with T2DM and the preventive effect of metformin on EC risk was not illustrated [49]. Tang et al. conducted a meta-analysis based on 11 studies and indicated metformin therapy are significantly improvement EC risk and prognosis of EC. However, this study with incomplete electronic searches and the result of recurrence of EC were not calculated [50]. This novel meta-analysis of metformin use for preventing and treating EC analyzed both risk and survival scenarios. The most comprehensive up-to-date relevant studies were included. The sample sizes of most studies were sufficiently large, and the studies were of high quality. The inclusion of participants from all parts of the world meant that the present study results should be generalizable to the general population. Most studies sufficiently adjusted for various clinicopathological confounding factors. Moreover, the role of metformin was specifically assessed among patients with diabetes.

Abundant preclinical in vitro and in vivo studies have reported the anticancer effect of metformin on various malignancies. Nevertheless, the exact molecular mechanisms remain unknown. Metformin may inhibit cancer stem cell–like subpopulations in cases of intraepithelial neoplasia [51]. Metformin may also prevent the conversion of epithelial cells into mesenchymal cells [52]. Several studies have reported that metformin can reverse endometrial hyperplasia [53, 54]. Thus, metformin may have multiple functions mediated through direct and indirect mechanisms [51]. The indirect effect is insulin dependent. Metformin helps control the circulating glucose level and improve insulin sensitivity. The direct effect is insulin independent. Metformin exerts its effects on tumor cells primarily through the adenosine 5′-monophosphate–activated protein kinase and phosphoinositide 3-kinase/protein kinase B/mammalian target of rapamycin signaling pathways [55]. Notably, these molecular targets are similar to the targets of current drugs, such as sorafenib and everolimus. A phase I clinical trial of 21 cases, including 4 patients with advanced EC, showed that the combination of temsirolimus and metformin was a promising treatment [56]. Metformin is nontoxic and may be extremely useful for enhancing the treatment efficacy of the targeted drugs [51]. Since metformin has been used for more than 50 years, its safety profile has been well established. Although it can cause potentially dangerous toxicity from lactic acidosis, the risk is mainly confined to patients aged more than 80 years, patients with alcohol abuse, or those who have comorbidities of renal, hepatic, or cardiac insufficiency [57].

This meta-analysis had several limitations. It only identified a small number of studies exploring the role of metformin for prevention and treatment. Further, stratified results according to individuals characteristics were not reported. Therefore, a subgroup or meta-regression analysis could not identify the sources of heterogeneity. Many of the included studies were retrospectively designed, which might have led to recall and selection bias, and those study are associated with low level of evidence. Furthermore, whether patients had taken different antidiabetic drugs before metformin administration could not be determined. Their glycemic control might also be inadequate. Moreover, this meta-analysis was based on observational data, which was associated with higher indication bias. Such as, more “healthier patients” always receive the best treatment. No randomized controlled trial has been conducted on patients with EC. Interestingly, a previous large randomized controlled trial that enrolled patients with gastrointestinal malignancies showed that metformin was helpful for the chemoprevention of colorectal cancer [58], but did not significantly improve the OS of patients with pancreatic cancer [59]. Most of the studies included in this meta-analysis did not report the effect of metformin dose or duration. Although most of the studies performed sufficient adjustment of variables, little information was available to evaluate the potential influence of other drugs such as aspirin or statins. Moreover, stratified analyses were not conducted based on study design and other patient characteristics, since a smaller number of cohorts were included. Therefore, this comprehensive meta-analysis just provided relative results on metformin use for EC prevention and treatment.

## Conclusions

In conclusion, a preventive effect of metformin on the development of EC was not observed in this meta-analysis. However, metformin was beneficial in improving the OS and reducing the relapse risk for patients with EC. More prospective long-term studies should be conducted to verify the findings of the present study.

## Additional files

Additional file 1: PRISMA Checklist. (DOC 65 kb)

Additional file 2: Newcastle–Ottawa scale for quality assessment of the included studies. (DOC 36 kb)

## Abbreviations

CI: Confidence interval
EC: Endometrial cancer
HR: Hazard ratio
OR: Odds ratio
OS: Overall survival
RFS: Recurrence-free survival
RR: Relative risk
