# Supplementary material for: Effect of metformin use on the risk and prognosis of endometrial cancer: a systematic review and meta-analysis
Source: BMC Cancer. 2018 Apr 18;18:438. doi: 10.1186/s12885-018-4334-5 (PMC5907461; doi:10.1186/s12885-018-4334-5)
Supplement: Supplementary file 2 — Newcastle–Ottawa scale for quality assessment of the included studies. (DOC 36 kb) [file 12885_2018_4334_MOESM2_ESM.doc]

**Additional file 2.** Newcastle–Ottawa scale for quality assessment of the included studies

| Study | Selection | Comparability | Outcome | NOS scale |
| --- | --- | --- | --- | --- |
| Endometrial cancer risk |  |  |  |  |
| Becker et al. (2013) | **** | ** | *** | 9 |
| Luo et al. (2014) | **** | ** | *** | 9 |
| Ko et al. (2015) | **** | ** | ** | 8 |
| Soffer et al. (2015) | **** | * | *** | 8 |
| Tseng et al. (2015) | **** | ** | *** | 9 |
| Franchi et al. (2016) | **** | ** | *** | 9 |
| Arima et al. (2017) | **** | ** | *** | 9 |
| Endometrial cancer survival |  |  |  |  |
| Ko et al. (2014) | *** | ** | *** | 8 |
| Nevadunsky et al. (2014) | **** | ** | *** | 9 |
| Lemanska et al. (2015) | **** | ** | *** | 9 |
| Ezewuiro et al. (2016) | *** | ** | *** | 8 |
| Hall et al. (2016) | **** | * | *** | 8 |
| Al Hilli et al. (2016) | **** | ** | *** | 9 |
| Seebacher et al. (2016) | **** | ** | *** | 9 |
